# An interpretable survival model for diffuse large B-cell lymphoma patients using a biologically informed visible neural network

**DOI:** 10.1016/j.csbj.2024.07.019

**Published:** 2024-07-24

**Authors:** Jie Tan, Jiancong Xie, Jiarong Huang, Weizhen Deng, Hua Chai, Yuedong Yang

**Affiliations:** a School of Computer Science and Engineering, Sun Yat-sen University, Guangzhou, China; b Guangzhou KingMed Center for Clinical Laboratory Co. Ltd., Guangzhou, China; c Key Laboratory of Machine Intelligence and Advanced Computing of MOE, Sun Yat-sen University, Guangzhou, China,; d School of Mathematics and Big Data, Foshan University, Foshan, China

**Keywords:** DLBCL, VNN, Survival model, Prognosis, Interpretable, Subtype

## Abstract

Diffuse large B-cell lymphoma (DLBCL) is the most common subtype of non-Hodgkin lymphoma (NHL) and is characterized by high heterogeneity. Assessment of its prognosis and genetic subtyping hold significant clinical implications. However, existing DLBCL prognostic models are mainly based on transcriptomic profiles, while genetic variation detection is more commonly used in clinical practice. In addition, current clustering-based subtyping methods mostly focus on genes with high mutation frequencies, providing insufficient explanations for the heterogeneity of DLBCL. Here, we proposed VNNSurv (https://bio-web1.nscc-gz.cn/app/VNNSurv), a survival model for DLBCL patients based on a biologically informed visible neural network (VNN). VNNSurv achieved an average C-index of 0.72 on the cross-validation set (HMRN cohort, n = 928), outperforming the baseline methods. The remarkable interpretability of VNNSurv facilitated the identification of the most impactful genes and the underlying pathways through which they act on patient outcomes. When only the 30 highest-impact genes were used as genetic input, the overall performance of VNNSurv improved, and a C-index of 0.70 was achieved on the external TCGA cohort (n = 48). Leveraging these high-impact genes, including 16 genes with low (<5 %) alteration frequencies, we devised a genetic-based prognostic index (GPI) for risk stratification and a subtype identification method. We stratified the patient group according to the International Prognostic Index (IPI) into three risk grades with significant prognostic differences. Furthermore, the defined subtypes exhibited greater prognostic consistency than clustering-based methods. Broadly, VNNSurv is a valuable DLBCL survival model. Its high interpretability has significant value for precision medicine, and its framework is scalable to other diseases.

## Graphical Abstract

**Figure:**
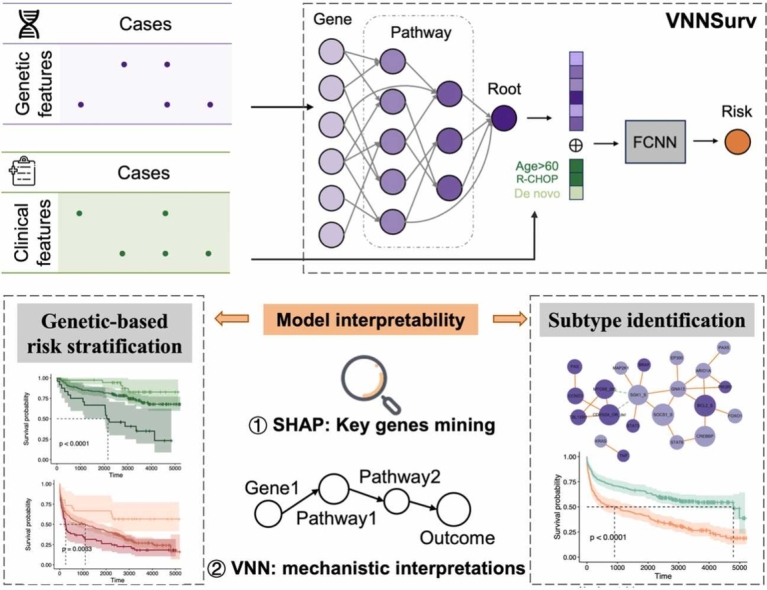

## 1 Introduction

Diffuse large B-cell lymphoma (DLBCL) is one of the most prevalent subtypes of non-Hodgkin lymphoma (NHL), accounting for approximately 30 %−50 % of all NHL cases [1]. DLBCL is highly heterogeneous, with substantial variations in clinical features, treatment responses, and survival risks observed among patients, posing a substantial challenge to clinical diagnosis and treatment [2], [3]. Consequently, the prognostic assessment and subtyping of DLBCL patients are clinically important.

The prognostic assessment of cancer patients has consistently remained a focal topic in the field of medical research, garnering considerable attention. Considering the high heterogeneity of DLBCL, a prognosis assessment of patients is often required to formulate suitable therapeutic schedules. Currently, prognosis evaluations of DLBCL patients are predominantly reliant on the traditional International Prognostic Index (IPI) or the revised IPI (R-IPI) [4], [5] that uses a few phenotypic characteristics to categorize patients into 3 to 4 risk grades, providing an insufficient explanation for the heterogeneity among patients. In 2002, a DLBCL outcome prediction model was constructed using gene expression profiling as a binary classifier to predict five-year survival in patients [6]. With the wide application of artificial intelligence technologies in the biomedical field, many machine learning methods based on genetic information have been employed for cancer risk prediction and survival analysis [7], [8], [9]. However, existing prognostic models developed for DLBCL are mostly based on gene expression profiles and traditional machine learning models[10], [11], [12]. Currently, clinical testing for DLBCL patients primarily relies on genetic variation detection, limiting the clinical application of models developed based on transcriptomic data. Therefore, the development of more precise and efficient prognostic assessment methods for DLBCL is urgently needed.

With the advancement of sequencing technologies and analysis methods, the molecular subtype classification of cancer has become the basis for prognosis assessment and treatment selection. Hence, the molecular subtyping of DLBCL has also gained increasing attention in recent years. In 2000, researchers defined two primary cell of origin (COO) subtypes based on differences in gene expression profiles: germinal center B-cell-like (GCB) and activated B-cell-like (ABC) [13], [14], [15]. However, the COO classification does not fully account for the heterogeneous responses and treatment outcomes in DLBCL patients [16]. This lack of explanation may be because gene expression profiles provide a phenotypic description of cancer rather than a genetic representation that directly addresses the tumorigenic mechanisms. As a result, an increasing number of studies have been dedicated to exploring the genetic drivers behind the heterogeneity of DLBCL [3]. Since 2018, DLBCL subtyping methods based on genetic features have emerged and matured. Several studies utilizing whole-exome sequencing, transcriptome sequencing, DNA copy number variation (CNV) analysis, and targeted resequencing data independently identified 4 to 7 distinct genetic subtypes of DLBCL with varied prognoses and treatment impacts [16], [17], [18], [19], [20]. However, despite some overlap between the genotypes included in these studies, there is still no widely accepted schematic for the definition of DLBCL genotypes. Additionally, these clustering-based methods often focus on high-frequency mutated genetic features, resulting in a considerable portion of patients being unclassified. Therefore, for the identification of DLBCL genotypes, more effective strategies that explain the heterogeneity of DLBCL as comprehensively as possible are required.

Although machine learning algorithms have achieved widespread application and recognition in the biomedical field [21], [22], they still face the specific challenge of lacking mechanistic interpretations [23], [24]. In 2018, the concept of visual machine learning was proposed, and subsequent visual neural networks (VNNs) provide a novel perspective for the mechanistic explanation of biomedical problems [24]. In a visual framework, neurons are mapped to biological entities such as genes, proteins, pathways, and cellular subsystems, with the connections between neurons representing biological relationships. Recently, biologically informed neural networks have been successfully applied in cancer diagnosis and drug response prediction [25], [26], [27], [28]. Moreover, the SHapley Additive exPlanations (SHAP) method offers a robust and consistent way to measure the impact of features in a machine learning model [29]. Numerous studies have effectively utilized SHAP to determine the most impactful factors in cancer prognosis models [30], [31]. Therefore, we anticipate that the combination of SHAP and a VNN to construct a prognostic model for DLBCL could aid in the further exploration of high-impact genes and the investigation of their underlying pathogenic mechanisms.

In this study, we developed VNNSurv, an interpretable prognostic model for DLBCL based on a VNN. Its remarkable interpretability harnessed through VNNs and SHAP has enabled the exploration of the driver genes and pathogenic mechanisms underlying DLBCL heterogeneity. Moreover, 30 high-impact genes derived from the model interpretability were successfully used to design new genetic-based risk stratification and subtype identification methods.

## 2 Materials and methods

### 2.1 Data preparation

The data used for model construction were obtained from a cohort comprising 928 DLBCL patients (denoted HMRN) reported by Stuart et al. in a related study [19]. Two external validation cohorts, the TCGA cohort (n = 48) and the BCCA cohort (n = 329) [32], [33], were included to assess the generalizability of the model. These datasets consisted of patients' basic information, clinical variables, and gene alterations, including single-nucleotide polymorphisms (SNPs) and CNVs. A total of 120 features from the HMRN cohort were utilized as inputs for the initial model construction, including 117 recurrent gene alterations (105 SNPs and 12 CNVs) and 3 potentially impactful clinical variables (age at diagnosis, R-CHOP treatment, and de novo DLBCL) [34]. We employed one-hot encoding to transform these features into a format conducive to model processing. Specifically, if a patient exhibited a particular gene alteration, the corresponding feature was assigned a value of 1; otherwise, it was set to 0. The three clinical variables were encoded as 1 if the patient's age at diagnosis was over 60 years, the patient received R-CHOP treatment, or the patient they had de novo DLBCL. Notably, due to differences in the technologies and the gene panel of targeted sequencing used for detection, some features were missing in the two external cohorts. When there were missing features, we uniformly assigned a default value (0 or 1) to all patients in that cohort. The gene alterations and clinical information for the three cohorts are available in Supplementary Material 2 at https://github.com/jie-tan/vnnsurv.

### 2.2 VNNSurv framework

**Fig. 1:**
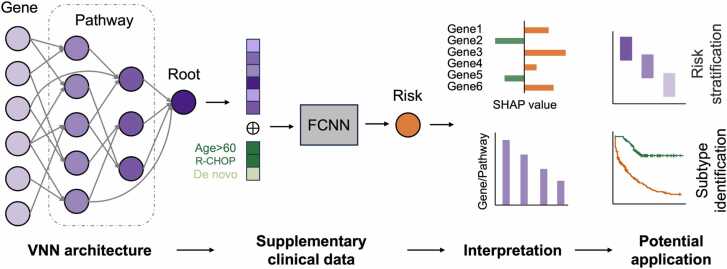
**Schematic representation of VNNSurv and its subsequent interpretation and application.** VNNSurv is a highly interpretable prognostic model based on a VNN architecture and the SHAP method. The gene alteration profiles of patients are first input into the VNN architecture, a gene and pathway-guided neural network, to extract and integrate information as genetic features. Combined with three key clinical attributes, these features are then pass to a fully connected neural network to derive the risk scores. The trained VNNSurv provides a quantitative approach to assess the impact of each gene and pathway on patient outcome and further aids in risk stratification and subtype identification.

VNNSurv consists of two main components (Fig. 1): the VNN architecture, which processes and represents the gene alteration profile, and a fully connected neural network (FCNN), which integrates genetic features with clinical variables.

To design a VNN architecture that simulates cell biological processes, we first downloaded the pathways gene set and pathways hierarchy relationship files from Reactome (https://reactome.org/) [35] to obtain valuable insights into gene-to-pathway and pathway-to-pathway relationships. Next, we constructed a gene and pathway-guided VNN architecture for the input of 117 genetic features (Fig. 1). The neurons in the input layer of the VNN were mapped to the 117 gene alterations, and the neurons in the hidden layers were mapped to the hierarchical pathways associated with these genes. As a sparse neural network, connections between neurons were established only when there was a biological association between the genes or the pathways they represent. Additionally, neurons representing pathways without parent pathways were connected to the root neuron. Notably, some neurons were skip-connected due to the non-strict hierarchical organization of pathways in Reactome. As a result, the VNN architecture encompassed 102 pathways distributed across seven layers. The root neuron had an output dimension of 283, which served as the embedding for the gene alteration profile. This VNN output was then concatenated with three clinical variables and input into a two-layer FCNN to generate the patient's survival risk.

Since there are currently no survival models specifically designed for DLBCL that utilize machine learning and genetic features, we devised two baseline methods for comparison, the FCNN and a Cox proportional hazards (CoxPH) model. CoxPH is one of the most widely used classical survival models. The FCNN has a similar number of hidden layers and neurons to those in VNNSurv. We adopted the same hyperparameter tuning strategy used in VNNSurv for the FCNN.

### 2.3 Model training

VNNSurv underwent a rigorous training process to achieve optimal performance. We employed the AdamW optimizer with fixed weight decay and applied dropout on both the VNN and FCNN layers to prevent overfitting. We conducted a 10-fold cross-validation to determine the optimal hyperparameters of the model, including the learning rate, weight decay, batch size, epochs, and dropout rate. The nonlinear proportional hazards loss function, a well-established choice in survival models, was utilized to update the model parameters [8]. The formula is as follows:

$$L(\theta )=\sum_{i\in D}\left({\hat{r}}_{\theta }\left(X_{i}\right)\;-\log(\sum_{j\in R(t_{i})}\exp({\hat{r}}_{\theta }(X_{j})))\right)$$

where θ is the current parameter of the model, ${\hat{r}}_{\theta }\left(X_{i}\right)$ and ${\hat{r}}_{\theta }\left(X_{j}\right)$ are the risk scores of the i-th and j-th patients under the current parameter, D is the set of patients who have observed the event (death), and $R(t_{i})$ is the set of patients who are still alive at the time of death of the i-th patient $t_{i}$.

### 2.4 Model evaluation

To evaluate model performance, we used the concordance index (C-index), which is widely used in survival models. The C-index ranges between 0 and 1, reflecting the consistency between the model's predicted risk scores and the actual observed survival times for pairs of patients. The formula is as follows:

$$C-\mathrm{index}=\;\frac{1}{N_{\mathrm{effect}}}\sum_{i\in D}\sum_{j\in R(t_{i})}I[{\hat{r}}_{i}>{\hat{r}}_{j}]$$

where $N_{\mathrm{effect}}$ refers to the number of effective patient pairs and ${\hat{r}}_{i}$and ${\hat{r}}_{j}$ represent the predicted survival risks for the i-th and j-th patients, respectively. “I” is an indicative function, which is set to 1 when ${\hat{r}}_{i}>{\hat{r}}_{j}$ is true and 0 otherwise.

### 2.5 Model interpretability

The interpretability of VNNSurv consists of two parts: a post hoc explanation based on SHAP and a visual explanation leveraging the VNN architecture. We utilized the Python package shap (V0.41.0) to calculate SHAP values for each input feature and each pathway in the hidden layer. The most commonly employed method for selecting significant features based on SHAP values involves calculating the sum of the absolute SHAP values across all samples. However, with this method, some low-frequency gene alterations might be overlooked due to their limited number. To better identify genetic features that significantly impact prognosis, including genes with low variation frequencies, only patients with specific alterations were included in the calculation of feature impact. The impact of the j-th genetic feature was calculated as follows:

$${\mathrm{Impact}}_{j}=\;\frac{1}{N_{j}}\sum_{i\in C_{j}}s_{\mathrm{ij}}$$

where $N_{j}$ represents the number of cases with a j-th feature of 1, $C_{j}$ represents the set of cases with a j-th feature of 1, and $s_{\mathrm{ij}}$ represents the SHAP value of the j-th feature of the i-th case.

Furthermore, we utilized multilayered Sankey diagrams to visualize the VNN structure, which could help in understanding how genes influence patient outcomes. In the Sankey diagram, the “source” in the first layer represents genetic features, and the “target” corresponds to the initial layer of pathways. In the subsequent layers, “source” represents child pathways, and “target” represents parent pathways. The width of each “source” and “target” was determined by their respective SHAP values. The width of the connections between “source” and “target” was determined by the proportion of the SHAP value of the “source” among all SHAP values at the “target” inputs.

### 2.6 Genetic-based risk stratification

We designed a genetic-based prognostic index (GPI) based on key genes and their impacts to complement the phenotype-based IPI. The GPI score for the i-th patient was calculated as follows:

$${\mathrm{score}}_{i}=\;\sum_{j\in F_{\mathrm{key}}}{f_{\mathrm{ij}}*\mathrm{Impact}}_{j}$$

where $F_{\mathrm{key}}$ represents the set of key genetic features, $f_{\mathrm{ij}}$ is the value (0 or 1) of the j-th feature for the i-th case, and ${\mathrm{Impact}}_{j}$ is the impact of the j-th feature. Then, this score was used for the refined risk stratification of patients at different IPI grades.

$${\mathrm{level}}_{i}=\left\{\begin{array}{c} \mathrm{level}-I,\mathrm{if}{\mathrm{score}}_{i}\leq t1\;\\ \mathrm{level}-\mathrm{II},\mathrm{if}{t1<s\mathrm{core}}_{i}\leq t2\;\\ \mathrm{level}-\mathrm{III},\mathrm{if}{s\mathrm{core}}_{i}>t2.\; \end{array}\right)$$

The thresholds t1 and t2 were determined by the significance of differences between patient risk at different levels.

### 2.7 Subtype identification

We performed three interaction analyses on the key genes to mine new DLBCL genotypes. These analyses included (1) exploring the interaction relationships among the proteins encoded by these genes using the STRING database [36], (2) identifying the co-occurrence and mutual exclusivity relationships between the gene alterations through Fisher's test and false discovery rate (FDR) correction, and (3) clarifying the frequency of the combinations of gene alterations by an UpSet plot.

### 2.8 Statistical analysis

Comparisons of the C-index between different methods and the survival curves between different groups of patients were conducted using Wilcoxon's rank-sum test and log-rank test in R 4.2.1, respectively. Fisher’s test and the FDR correction were used to test the co-occurrence and mutual exclusivity relationships between genetic variants. For all the statistical tests, p < 0.05 was considered to indicate statistical significance. Asterisks indicate the significance levels of p values: ns, not significant; *p < 0.05; **p < 0.01; ***p < 0.001; ****p < 0.0001.

## 3 Results

### 3.1 The performance of VNNSurv

**Fig. 2:**
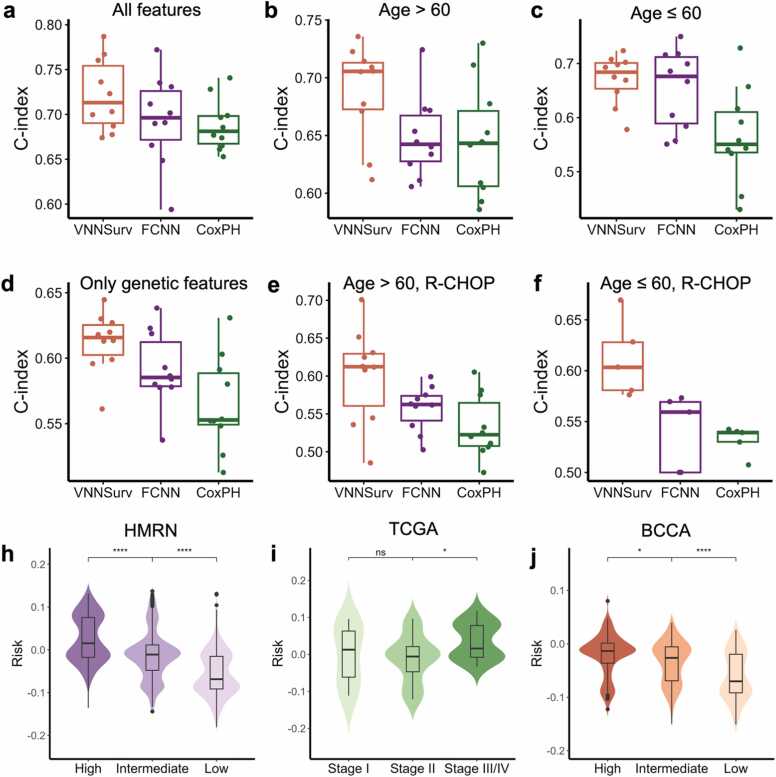
**Performance of VNNSurv and comparison methods.** (a-f) C-index distributions of VNNSurv, HMRN, and CoxPH on the cross-validation sets. FCNN: fully connected neural network, CoxPH: Cox proportional hazards. (a-c) Both genetic and clinical features are used as inputs; (a) all cases are included, (b) only cases with an age at diagnosis > 60 years old are included, (c) only cases with an age at diagnosis ≤ 60 years old are included. (d-f) Only genetic features are used as inputs; (d) all cases are included, (e) only cases with an age at diagnosis > 60 years old and receiving R-CHOP treatment are included, (f) only cases with an age at diagnosis ≤ 60 and receiving R-CHOP treatment are included. (h-j) Distribution of risk scores predicted by VNNSurv across various IPI grades or disease stages for the HMRN cohort (h), TCGA cohort (i), and BCCA cohort (j).

First, the performance of VNNSurv was assessed on the 10-fold cross-validation set and compared with baseline methods. The cross-validation set was randomly split from 928 patients in the HMRN cohort. When utilizing all 120 features as input (Fig. 2a), VNNSurv (C-index: 0.72 ± 0.05) outperformed FCNN (0.69 ± 0.05) and CoxPH (0.69 ± 0.03). To validate the ability of VNNSurv to assess risk differences in patients at similar ages, we divided the HMRN cohort into two age subsets and constructed cross-validation sets. VNNSurv exhibited the least reduction in the C-index within the subsets compared with that tested on the full HMRN cohort, whereas the performance of CoxPH decreased significantly (Fig. 2b-c). Furthermore, we evaluated the models using only the 117 genetic features as input, which provides a direct assessment of the ability of the VNN to encode genetic information. The C-indices of the three methods decreased as expected, but the advantage of VNNSurv over the other two methods increased (Fig. 2d). Given that DLBCL patients receive the standard treatment regimen R-CHOP, we further divided treated patients into different age subsets. We re-evaluated the three models using only genetic features as input and found that the results of VNNSurv remained the most stable (Fig. 2e-f). These results indicate that deep neural networks possess more robust prognostic assessment capabilities than the CoxPH model, and the sparsely connected VNN architecture exhibits stronger representation abilities of genetic features than ordinary FCNNs.

Subsequently, we retrained VNNSurv with the entire HMRN cohort and assessed its generalizability using two external cohorts. The risk scores predicted by VNNSurv for high-risk patients were significantly greater than those for patients with other disease stages or IPI grades (Fig. 2h-j). The C-indices for the TCGA and BCCA cohorts were 0.65 and 0.62, respectively. As mentioned earlier, the available information in the two external cohorts was somewhat inconsistent with that of the HMRN cohort. We evaluated the consistency of clinical variables, SNPs, and CNVs between the external cohorts and the HMRN cohort (Table S1). The TCGA cohort, which exhibited better performance, demonstrated greater consistency, which was in line with our expectations. Then, we retrained the model using the intersection of features from the HMRN and BCCA cohorts and found that the model's C-index decreased only slightly (Table S2). Therefore, the evaluation results on the external cohorts verified the predictive and generalization abilities of VNNSurv.

### 3.2 The interpretability of VNNSurv

**Fig. 3:**
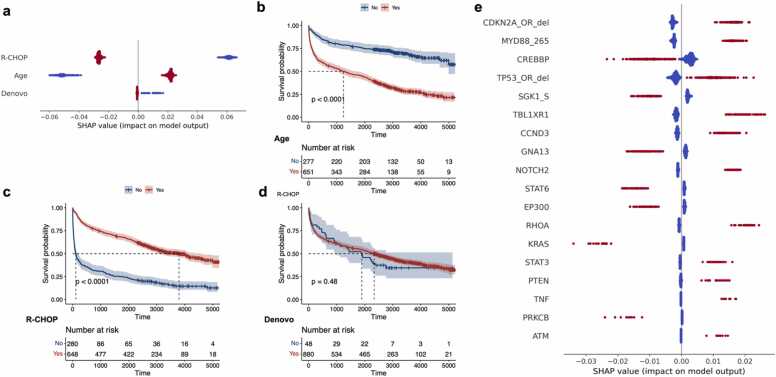
**Overview of VNNSurv’s interpretability based on SHAP values.** (a) Summary plot of the SHAP values for three clinical variables. (b), (c), and (d) Survival curve plots of patients grouped according to clinical features: age at diagnosis (b), R-CHOP treatment (c), and de novo DLBCL (d). (e) Summary plot of the SHAP values for the top 18 high-impact genetic features. Red indicates cases with a feature value of 1, while blue represents cases with a feature value of 0.

Next, we utilized the interpretability results of the trained VNNSurv to mine driver genes and investigate the underlying pathogenic mechanisms. We first examined the impacts of three clinical variables using SHAP values. Receiving R-CHOP treatment and being diagnosed at an age younger than 60 years were found to significantly reduce patients' risk, while de novo cancer did not appear to be a high-impact factor (Fig. 3a). These findings were consistent with the conclusions obtained by the differences between the survival curves of patients grouped based on these three variables (Fig. 3b-d). Therefore, these results validate that VNNSurv can accurately identify crucial prognostic factors based on the SHAP values.

Next, we further mined high-impact genetic features using the SHAP values. The SHAP values of each genetic feature of every patient were calculated (Supplementary Material 2). Utilizing the calculations introduced in the Methods section, we computed the prognostic impact of each genetic feature. The distribution of SHAP values for the top 18 high-impact genetic features is illustrated in Fig. 3e. Several genes, such as *MYD88*, *TP53*, *SGK1*, and *NOTCH2*, which have been used as DLBCL subtype classification criteria in previous studies [16], [37], [38], were determined to have relatively high impact. The results demonstrated that the *MYD88* L265P mutation is a poor prognostic factor, which has been verified in multiple studies [16], [19], [37]. Moreover, VNNSurv identified several low-frequency but high-impact gene alterations that are overlooked by existing DLBCL subtype definition schemes. For instance, *KRAS* and *RHOA* have been reported as potential genetic drivers of DLBCL but have not been used in relevant applications [39], [40], [41]. The results revealed that their absolute impact values were the highest among all genes, further highlighting their potential for application in DLBCL prognostic assessment, despite their relatively low alteration frequencies. Considering that current DLBCL genotypes only classify a portion of cases and fail to fully explain the heterogeneity of DLBCL, the identification of driver genes based on the interpretability of VNNSurv might have the potential to augment the definition of novel DLBCL subtypes.

**Fig. 4:**
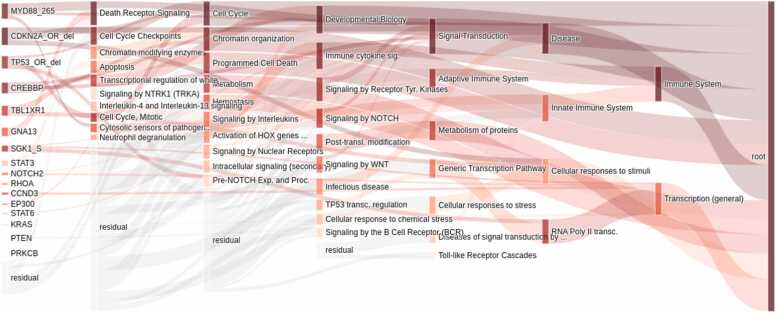
**Illustration of VNN architecture after model construction.** Node lengths are determined by their respective mean SHAP values, while the widths of edges between nodes are determined by the mean SHAP values from the source node to the target node.

Furthermore, we leveraged the SHAP values and visualized the VNN architecture to facilitate the exploration of the pathogenic mechanisms of high-impact genes. The VNN architecture was visualized to illustrate how these high-impact genes act on patient outcomes by individual pathways (Fig. 4). To make the visualization clearer, we removed some of the less important connections between genes and pathways. For example, the *MYD88* L265P mutation affects patient risk through the “death receptor signaling”, “cell cycle”, “cytosolic sensors of pathogen-associated DNA”, “innate immune system” and “disease” pathways. Through these visualized pathways, researchers can conduct more targeted studies on the pathogenic mechanisms of disease-driving genes.

### 3.3 Risk stratification based on high-impact genes

**Fig. 5:**
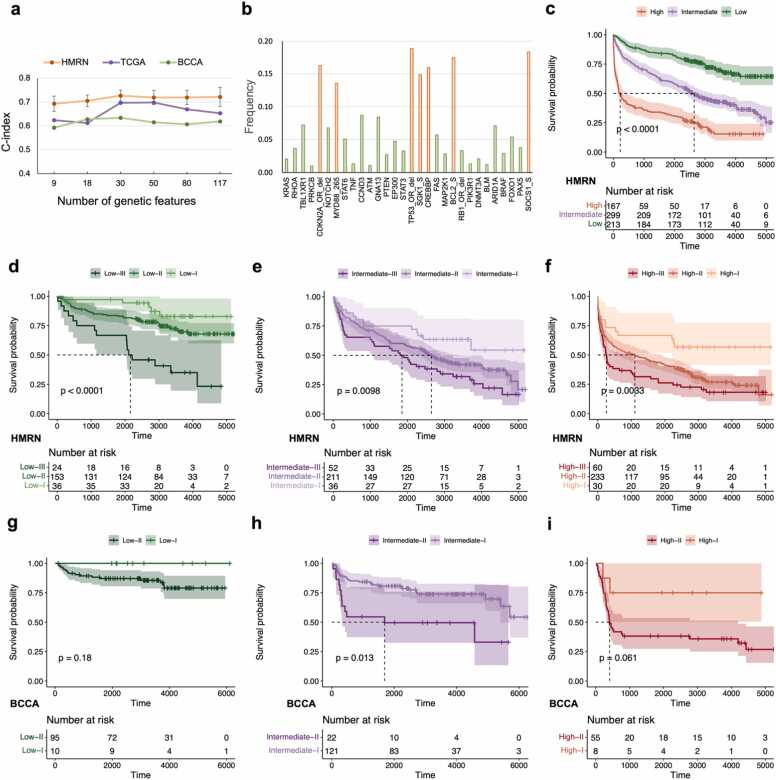
**The potential of high-impact genetic features in prognosis prediction and risk stratification.** (a) Cross-validation on the HMRN cohort and external validation on the TCGA and BCCA cohorts using different numbers of high-impact genetic features. (b) The alteration frequencies of the top 30 genes. (c) Survival curves of patients at different IPI grades in the HMRN cohort. (d-f) Refined risk stratification of patients with low-risk (d), intermediate-risk (e), and high-risk (f) IPI grades in the HMRN cohort based on the GPI. (g-i) Refined risk stratification of patients with low-risk (g), intermediate-risk (h), and high-risk (i) IPI grades in the BCCA cohort based on the GPI.

Subsequently, we explored the potential applications of high-impact genes in prognosis assessment and risk stratification. We gradually removed the least impactful genes based on the ranking of their impact values and retrained VNNSurv with the new feature sets. As the number of retained features decreased, the C-index on the cross-validation set fluctuated slightly, while the performance on the two external validation sets, especially the TCGA cohort, increased significantly (Fig. 5a). Notably, the overall performance of VNNSurv was optimized (HMRN: 0.73, TCGA: 0.70, BCCA: 0.63) when modeling with only the top 30 genetic features (Fig. 3e and Fig. S1) and three clinical variables. With a further reduction in the number of features, the model exhibited a significant decrease in performance across all three cohorts. This result suggested that more features in the prognostic model do not necessarily lead to better performance. Hence, we also released a version of VNNSurv constructed based on the top 30 genetic features that users can select. This feature reduction further decreased the detection cost required for VNNSurv and greatly enhanced its clinical application potential.

Among these 30 genes, 7 were frequently altered (with frequencies greater than 13.58 %, indicated by the red bars in Fig. 5b), and they have all been used previously for the definition of DLBCL subtypes [16], [19]. The remaining genetic features with low alteration frequencies (indicated by the green bars in Fig. 5b) have been less extensively studied, especially the 16 genes with an alteration frequency of less than 5 %. Therefore, we anticipated that these 30 genetic features selected by VNNSurv hold considerable practical value in improving the current risk stratification and subtype classification schemes, addressing their insufficient explanation of the heterogeneity among DLBCL patients.

These 30 high-impact genes were subsequently used for the genetic-based risk stratification of DLBCL patients. The patients in the HMRN cohort who had been classified as low, intermediate, or high risk grades based on the IPI (Fig. 5c) were further stratified into three risk levels using the GPI scores as described in the Methods section. The two thresholds for stratification were −0.015 and 0.025. We identified 24 patients at relatively higher risk (Low-III in Fig. 5d, median survival time ∼2000 days) among patients in a low IPI grade. Furthermore, patients with a relatively lower risk (intermediate-I in Fig. 5e and high-I in Fig. 5f, median survival time >5000 days) were also distinguished among patients in intermediate and high IPI grades.

To validate the ability of the GPI to distinguish patient risk, we further stratified the patients from the BCCA cohort. Considering the stratification capability of GPI scores for the HMRN cohort, we only performed a two-level stratification for the BCCA cohort. As shown in Fig. 5g-i, the GPI also demonstrated the ability to further stratify patients with the same IPI grade according to risk, although the significance decreased due to the reduced sample size. Since there were only 42 patients with disease stages in the TCGA cohort (Fig. S2b), we did not use this cohort to verify the validity of the GPI. These results suggested that the genetic-based risk stratification method achieved through these high-impact genes can serve as a valuable complement to the phenotypic-based IPI, allowing for a finer delineation of patient risk levels, especially for patients for whom risk stratification is challenging with only phenotypic characteristics.

### 3.4 Subtype definition based on high-impact genes

**Fig. 6:**
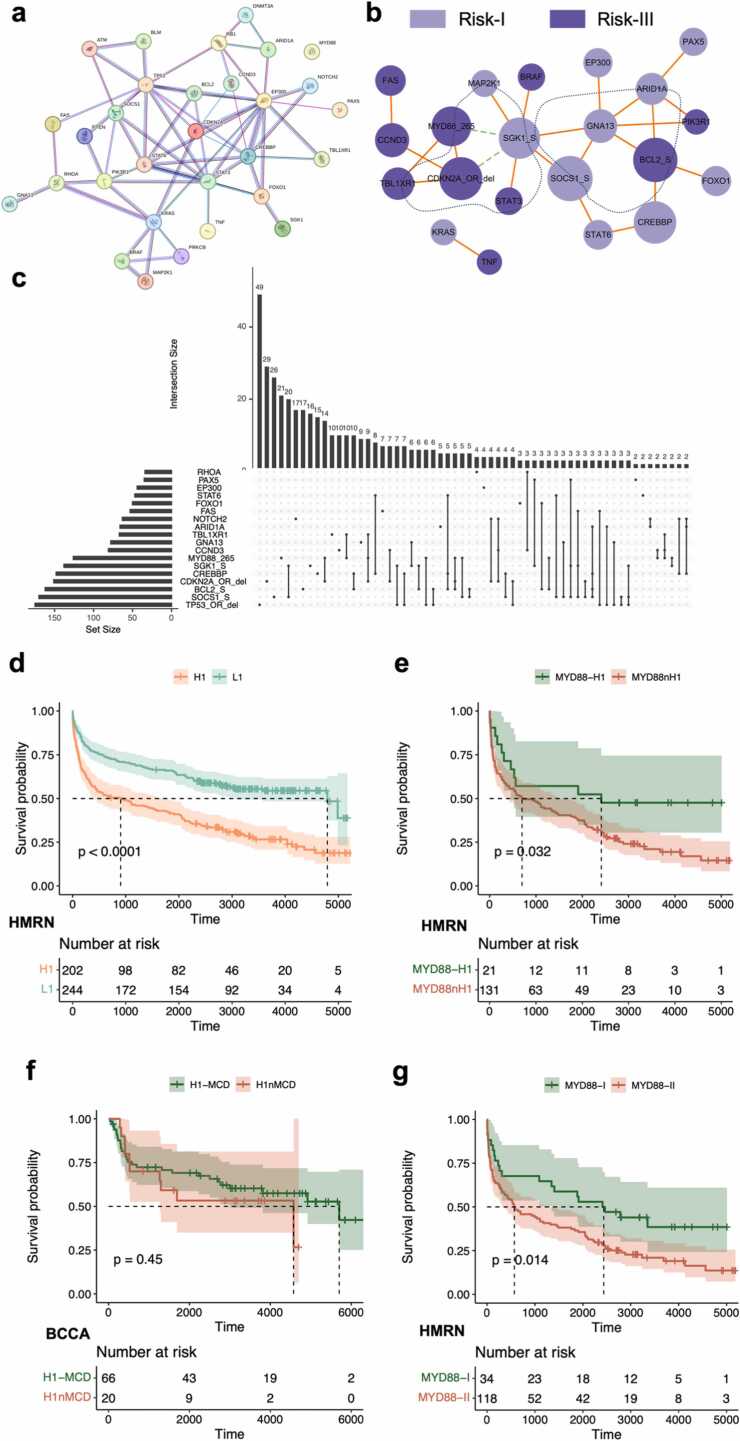
**Identification of new genetic subtypes and sub-subtypes of DLBCL based on interactions between high-impact genes.** (a) Protein—protein interaction network of the proteins encoded by these genes based on the STRING database. The specific meanings of the various colored edges can be found on the STRING website. (b) Co-occurrence and mutual exclusivity between pairs (FDR-corrected p value <0.05) of gene alterations, represented by solid red lines and dashed green lines, respectively. Pale purple and deep purple represent non-poor prognostic factors (risk-I, impact<0) and poor prognostic factors (risk-III, impact>0), respectively, and the circle size is positively correlated with the frequency of gene alterations. (c) UpSet plot illustrating the intersections among high-impact gene alterations. (d) Survival curves for the newly defined H1 and L1 subtypes. (e) Survival curves for HMRN patients in the intersection and difference sets of the H1 and MYD88 subtypes. (f) Survival curves for BCCA patients in the intersection and difference sets of the H1 and MCD subtypes. (g) Survival curves of sub-subtypes distinguished according to the SHAP value ranking of the feature *MYD88* L265P.

The 30 high-impact genes were further utilized for the identification of DLBCL genotypes. We used the three methods introduced in the Methods section to analyze the interactions between these genes (Fig. 6a-c). Considering these interaction patterns and the risk levels of gene alterations, we defined two main DLBCL genetic subtypes, the high-risk H1 subtype characterized by *MYD88* L265P, *CDKN2A* and *TBL1XR1* alterations and the low-risk L1 subtype characterized by *SGK1*, *SOCS1* and *GNA13* alterations. The survival curve for the H1 subtype was significantly lower than that for the L1 subtype (Fig. 6d). Among the genes, *TBL1XR1* and *GNA13*, which exhibited relatively low mutation frequencies, were not used for subtype definition in the HMRN study. In addition, the most frequently mutated gene, *TP53*, did not exhibit any co-occurrence relationships, and the SHAP values of TP53 varied significantly among patients (Fig. 3e). Hence, although previous studies have used *TP53* as a basis for DLBCL subtype definition [16], [20], we believe that it may not be suitable as a separate criterion for subtyping. Furthermore, *NOTCH2* and *RHOA* not only exhibited weak relationships with other gene alterations but also had relatively consistent SHAP values among patients, suggesting their potential to serve as standalone criteria for subtype classification. In addition, the genes *KRAS* and *TNF* also have the potential for joint use in subtype definition.

Considering that high-risk patients often require more attention, we conducted an in-depth analysis of the subtype characterized by *MYD88* mutations. The main altered genes in the *MYD88* subtype defined by HMRN study included *MYD88*, *PIM1*, *CD79B*, *ETV6*, and *CDKN2A* [19], and the MCD subtype defined by LymphGen study is characterized by *MYD88* and *CD79B* alterations [16], which differ somewhat from the H1 subtype we defined. To elucidate the differences among patients in these subtypes, we compared the survival curves of patients in the intersection and difference sets of the *MYD88* and H1 subtypes in the HMRN cohort (Fig. 6e) and of patients in the intersection and difference sets of the MCD and H1 subtypes in the BCCA cohort (Fig. 6f). The results indicated that patients who were concurrently labeled with the H1 and MYD88 subtypes exhibited a worse prognosis. In other words, the prognostic consistency of the H1 subtype defined by VNNSurv was greater than that of the MYD88 subtype defined using clustering methods. Moreover, there was no significant difference in prognosis between the MCD and H1 subtypes, although the number of patients with the H1 subtype was considerably greater than that with the MCD subtype. This finding suggested that the definition criteria for the H1 subtype can identify more patients with a risk similar to that of MCD, addressing the issue that the existing subtype classification scheme does not cover all patients.

To clarify the origin of the internal heterogeneity within *MYD88*-related subtypes, we examined the SHAP values of the *MYD88* L265P alteration across different patients. We found that patients with the same key genetic variation may exhibit significant differences in the ranking of its impact (Fig. S3), which can be attributed to different genetic backgrounds. Based on whether the absolute SHAP value of the *MYD88* L265P alteration for a patient ranked in the top five among all genetic features, we redefined two sub-subtypes. Patients in the MYD88-II subgroup had a worse prognosis than those in the MYD88-I subgroup (Fig. 6g).

These results suggested that subtype identification from effect (risk) to cause (subtype) based on the interpretability of VNNSurv was superior to traditional clustering-based methods. This model-driven approach can define DLBCL subtypes with a stronger correlation to patient prognosis and identify sub-subtypes in which patients' risk levels are more consistent.

## 4 Discussion

In this study, we innovatively developed an interpretable survival model for DLBCL patients based on the VNN architecture. VNNSurv is a valuable DLBCL survival model, and its interpretability has encouraged us to delve into the genetic drivers and pathogenic mechanisms of DLBCL heterogeneity. Furthermore, VNNSurv requires only 30 high-impact genetic features and three easily obtainable clinical variables for prediction. Hence, it is more cost-effective than current mainstream prognostic models based on transcriptomic or multi-omics data. Overall, VNNSurv exhibits great potential for clinical applications. As a successful demonstration, its methodology can be adapted for research in other cancers in the future.

Notably, we conducted two applications based on model interpretability. First, we devised the GPI using high-impact genetic features selected by VNNSurv and implemented genetic-based risk stratification. This method allowed us to effectively categorize patients with varying prognoses among patients with the same IPI grade. Since IPI rely solely on several phenotypic characteristics, this genetic-based method can serve as a valuable complement. Second, we further explored DLBCL subtype definitions based on high-impact genes. As existing clustering-based subtype identification methods do not directly consider patient prognosis, our model-driven approach started with patient prognosis risk (effect) to identify the subtypes (cause), ensuring a greater correlation between the identified subtypes and prognosis. As a result, new subtypes with more uniform prognoses were identified, and sub-subtypes with significant prognostic differences were classified. Given that existing risk stratification and subtype identification methods are still limited in their explanation of the heterogeneity of DLBCL, the application of VNNSurv has important implications for improving this research gap.

However, there are limitations in this work. First, due to data limitations, the genetic information used in VNNSurv covers only a subset of gene mutations and CNVs, which may have led us to overlook some crucial prognostic factors. Moreover, as a highly heterogeneous cancer, DLBCL has been shown to exhibit population differences [42], [43]. Since the data we utilized for modeling came from three study, the identified risk factors and subtypes might be specific to certain populations. Additionally, for the VNN structure adopted by VNNSurv, we incorporated only gene and pathway information. In the future, we can explore integrating more biological entities into the network structure to enhance the model's performance.

In addition, we also recognize that the assistance provided by prognosis assessment in guiding clinical treatment strategies is limited. Currently, the standard chemotherapy regimen for DLBCL treatment is R-CHOP. However, approximately 40 % of patients still do not achieve remission or experience relapse after R-CHOP treatment [44], [45], [46]. Early identification of relapsed and refractory patients is clinically important for the successful treatment of DLBCL patients. Unfortunately, there is currently no mature method available to assess whether DLBCL patients exhibit resistance to standard R-CHOP treatment. Therefore, we are planning to develop another model to address this identification in the future, which will contribute to the resolution of challenges in the diagnosis and treatment of DLBCL.

## CRediT authorship contribution statement

**Jie Tan:** Conceptualization, Data curation, Formal analysis, Methodology, Software, Validation, Visualization, Writing – original draft, Writing – review & editing. **Yuedong Yang:** Conceptualization, Formal analysis, Funding acquisition, Methodology, Project administration, Software, Supervision, Visualization, Writing – review & editing. **Jiancong Xie:** Formal analysis, Methodology, Software, Visualization, Writing – review & editing. **Jiarong Huang:** Formal analysis, Visualization, Writing – review & editing. **Weizhen Deng:** Formal analysis, Visualization, Writing – review & editing. **Hua Chai:** Formal analysis, Funding acquisition, Writing – review & editing.

## Declaration of Competing Interest

None declared.

## References

[1] Swerdlow S.H., Campo E., Pileri S.A. (2016). The 2016 revision of the World Health Organization classification of lymphoid neoplasms. Blood.

[2] Zhang J., Grubor V., Love C.L. (2013). Genetic heterogeneity of diffuse large B-cell lymphoma. Proc Natl Acad Sci USA.

[3] Reddy A., Zhang J., Davis N.S. (2017). Genetic and functional drivers of diffuse large B cell lymphoma. Cell.

[4] Sehn L.H., Berry B., Chhanabhai M. (2007). The revised International Prognostic Index (R-IPI) is a better predictor of outcome than the standard IPI for patients with diffuse large B-cell lymphoma treated with R-CHOP. Blood.

[5] Zhou Z., Sehn L.H., Rademaker A.W. (2014). An enhanced International Prognostic Index (NCCN-IPI) for patients with diffuse large B-cell lymphoma treated in the rituximab era. Blood.

[6] Shipp M.A., Ross K.N., Tamayo P. (2002). Diffuse large B-cell lymphoma outcome prediction by gene-expression profiling and supervised machine learning. Nat Med.

[7] Cheng W.-Y., Yang T.-H.O., Anastassiou D. (2013). Development of a prognostic model for breast cancer survival in an open challenge environment. Sci Transl Med.

[8] Katzman J.L., Shaham U., Cloninger A. (2018). DeepSurv: personalized treatment recommender system using a Cox proportional hazards deep neural network. BMC Med Res Method.

[9] Chai H., Zhou X., Zhang Z. (2021). Integrating multi-omics data through deep learning for accurate cancer prognosis prediction. Comput Biol Med.

[10] Mosquera Orgueira A., Díaz Arias J.Á., Cid López M. (2020). Improved personalized survival prediction of patients with diffuse large B-cell Lymphoma using gene expression profiling. BMC Cancer.

[11] Dong H., Wang Q., Zhang G. (2020). OSdlbcl: an online consensus survival analysis web server based on gene expression profiles of diffuse large B‐cell lymphoma. Cancer Med.

[12] Merdan S., Subramanian K., Ayer T. (2021). Gene expression profiling-based risk prediction and profiles of immune infiltration in diffuse large B-cell lymphoma. Blood Cancer J.

[13] Alizadeh A.A., Eisen M.B., Davis R.E. (2000). Distinct types of diffuse large B-cell lymphoma identified by gene expression profiling. Nature.

[14] Rosenwald A., Wright G., Chan W.C. (2002). The use of molecular profiling to predict survival after chemotherapy for diffuse large-B-cell lymphoma. N Engl J Med.

[15] Wright G., Tan B., Rosenwald A. (2003). A gene expression-based method to diagnose clinically distinct subgroups of diffuse large B cell lymphoma. Proc Natl Acad Sci USA.

[16] Wright G.W., Huang D.W., Phelan J.D. (2020). A probabilistic classification tool for genetic subtypes of diffuse large B cell lymphoma with therapeutic implications. Cancer Cell.

[17] Schmitz R., Wright G.W., Huang D.W. (2018). Genetics and pathogenesis of diffuse large B-cell lymphoma. N Engl J Med.

[18] Chapuy B., Stewart C., Dunford A.J. (2018). Molecular subtypes of diffuse large B cell lymphoma are associated with distinct pathogenic mechanisms and outcomes. Nat Med.

[19] Lacy S.E., Barrans S.L., Beer P.A. (2020). Targeted sequencing in DLBCL, molecular subtypes, and outcomes: a Haematological Malignancy Research Network report. Blood.

[20] Shen R., Fu D., Dong L. (2023). Simplified algorithm for genetic subtyping in diffuse large B-cell lymphoma. Sig Transduct Target Ther.

[21] Shah P., Kendall F., Khozin S. (2019). Artificial intelligence and machine learning in clinical development: a translational perspective. npj Digit. Med.

[22] Haug C.J., Drazen J.M. (2023). Artificial intelligence and machine learning in clinical medicine, 2023. N Engl J Med.

[23] Lin C., Jain S., Kim H. (2017). Using neural networks for reducing the dimensions of single-cell RNA-Seq data. Nucleic Acids Res.

[24] Yu M.K., Ma J., Fisher J. (2018). Visible machine learning for biomedicine. Cell.

[25] Ma J., Yu M.K., Fong S. (2018). Using deep learning to model the hierarchical structure and function of a cell. Nat Methods.

[26] Kuenzi B.M., Park J., Fong S.H. (2020). Predicting drug response and synergy using a deep learning model of human cancer cells. Cancer Cell.

[27] Elmarakeby H.A., Hwang J., Arafeh R. (2021). Biologically informed deep neural network for prostate cancer discovery. Nature.

[28] Ghosh Roy G., Geard N., Verspoor K. (2022). MPVNN: Mutated Pathway Visible Neural Network architecture for interpretable prediction of cancer-specific survival risk. Bioinformatics.

[29] Lundberg S.M., Lee S.-I. (2017). A Unified Approach to Interpreting Model Predictions. Proceedings of the 31st International Conference on Neural Information Processing Systems.

[30] Li R., Shinde A., Liu A. (2020). Machine learning–based interpretation and visualization of nonlinear interactions in prostate cancer survival. JCO Clin Cancer Inform.

[31] Moncada-Torres A., Van Maaren M.C., Hendriks M.P. (2021). Explainable machine learning can outperform Cox regression predictions and provide insights in breast cancer survival. Sci Rep.

[32] Ennishi D., Jiang A., Boyle M. (2019). Double-hit gene expression signature defines a distinct subgroup of germinal center B-cell-like diffuse large B-cell lymphoma. J Clin Oncol.

[33] Ennishi D., Takata K., Béguelin W. (2019). Molecular and genetic characterization of MHC deficiency identifies EZH2 as therapeutic target for enhancing immune recognition. Cancer Discov.

[34] Coiffier B., Thieblemont C., Van Den Neste E. (2010). Long-term outcome of patients in the LNH-98.5 trial, the first randomized study comparing rituximab-CHOP to standard CHOP chemotherapy in DLBCL patients: a study by the Groupe d′Etudes des Lymphomes de l′Adulte. Blood.

[35] Gillespie M., Jassal B., Stephan R. (2022). The reactome pathway knowledgebase 2022. Nucleic Acids Res.

[36] Szklarczyk D., Gable A.L., Nastou K.C. (2021). The STRING database in 2021: customizable protein–protein networks, and functional characterization of user-uploaded gene/measurement sets. Nucleic Acids Res.

[37] Ngo V.N., Young R.M., Schmitz R. (2011). Oncogenically active MYD88 mutations in human lymphoma. Nature.

[38] Gao J., Sidiropoulou E., Walker I. (2021). *SGK1* mutations in DLBCL generate hyperstable protein neoisoforms that promote AKT independence. Blood.

[39] Newsam A., Goretsky Y., Roberts E. (2023). Abstract 1771: characterization of RHOA inactivation as a driver of CAR-T therapy resistance in diffuse large B-cell lymphoma. Cancer Res.

[40] Nagel P.D., Feld F.M., Weissinger S.E. (2013). Absence of BRAF and KRAS hotspot mutations in primary mediastinal and other diffuse large B-cell lymphoma. Blood.

[41] Zhang J., Reddy A., Davis N. (2017). Integrative analysis of 1001 diffuse large B cell lymphoma identifies novel oncogenic roles for Rhoa. Blood.

[42] Williams M.H., Williams R.A., Blaize J.P. (2020). The impact of race and ethnicity on diffuse large B-cell lymphoma (DLBCL) outcomes within the veterans health administration (VHA). Blood.

[43] Blansky D., Fazzari M., Mantzaris I. (2021). Racial and ethnic differences in diffuse large B-cell lymphoma survival among an underserved, urban population. Leuk Lymphoma.

[44] Wilson W.H. (2006). Drug resistance in diffuse large B-cell lymphoma. Semin Hematol.

[45] He M.Y., Kridel R. (2021). Treatment resistance in diffuse large B-cell lymphoma. Leukemia.

[46] Zhang J., Gu Y., Chen B. (2023). Drug-resistance mechanism and new targeted drugs and treatments of relapse and refractory DLBCL. CMAR.

