# Supplementary material for: An interpretable survival model for diffuse large B-cell lymphoma patients using a biologically informed visible neural network
Source: Comput Struct Biotechnol J. 2024 Jul 24;24:523–32. doi: 10.1016/j.csbj.2024.07.019 (PMC11357880; doi:10.1016/j.csbj.2024.07.019)
Supplement: Supplementary file 1 — Supplementary material [file mmc1.docx]

**Appendix for** **“Interpretable survival model for** **diffuse large B-cell lymphoma with** **biologically informed visible neural network”**


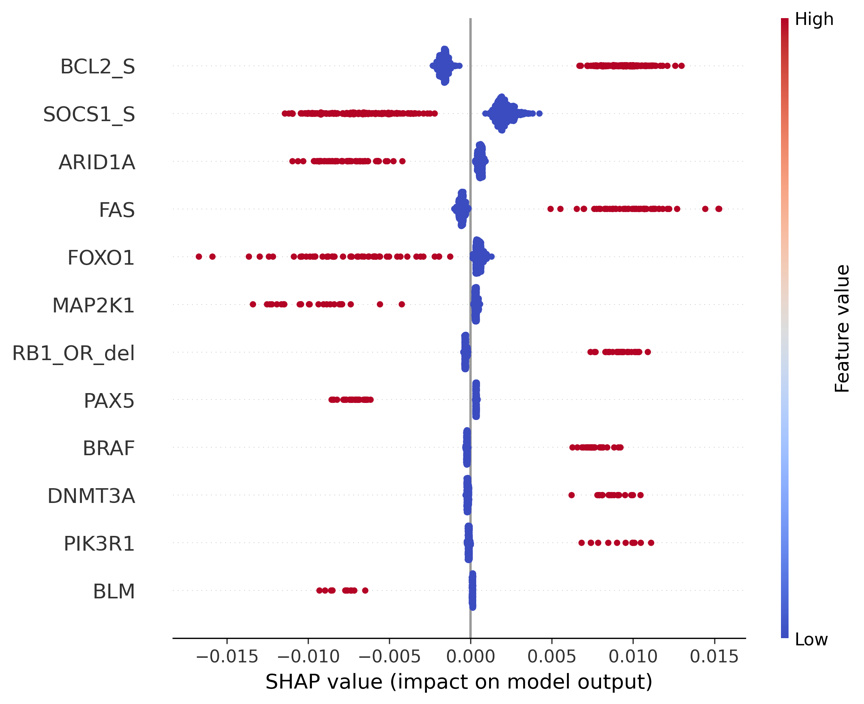


Figure S1. Summary plot of the SHAP values for the top 19~30 high-impact genetic features. Red indicates cases with a feature value of 1, while blue represents cases with a feature value of 0.

Table S1. Consistency of features between external cohorts and HMRN

| Features | Age | R-CHOP | De novo | SNPs (105) | CNVs (12) |
| --- | --- | --- | --- | --- | --- |
| TCGA | $\surd$ | $\surd$ | $\times$ | 84 (85%) | 12 (100%) |
| BCCA | $\surd$ | $\surd$ | $\surd$ | 61 (58%) | 12 (100%) |

Table S2. C-indices of the model retrained with intersection of HMRN and BCCA features

| Data | CV1 | CV2 | CV3 | CV4 | CV5 | CV6 |
| --- | --- | --- | --- | --- | --- | --- |
| C-index | 0.71 | 0.72 | 0.77 | 0.71 | 0.68 | 0.74 |
| Data | CV7 | CV8 | CV9 | CV10 | Mean | BCCA |
| C-index | 0.75 | 0.69 | 0.70 | 0.60 | 0.72 | 0.62 |


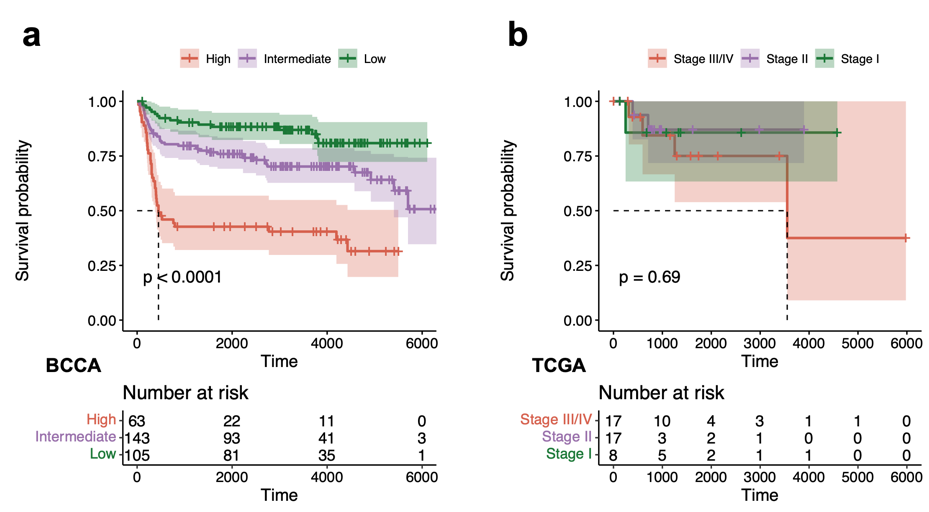


Figure S2. Survival curves of cases with different IPI risk grades and disease stages in the BCCA cohort (a) and the TCGA cohort (b).


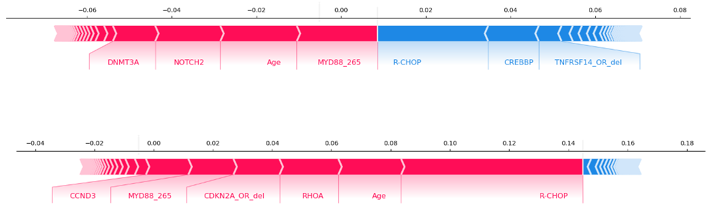


Figure S3. Variation in impacts of the same gene mutation across different cases.
